# Skeletal Muscle Fascicle Arrangements Can Be Reconstructed Using a Laplacian Vector Field Simulation

**DOI:** 10.1371/journal.pone.0077576

**Published:** 2013-10-25

**Authors:** Hon Fai Choi, Silvia S. Blemker

**Affiliations:** 1 Department of Mechanical & Aerospace Engineering, University of Virginia, Charlottesville, Virginia, United States of America; 2 Department of Biomedical Engineering, University of Virginia, Charlottesville, Virginia, United States of America; Stem Cell Research Institute, Belgium

## Abstract

Skeletal muscles are characterized by a large diversity in anatomical architecture and function. Muscle force and contraction are generated by contractile fiber cells grouped in fascicle bundles, which transmit the mechanical action between origin and insertion attachments of the muscle. Therefore, an adequate representation of fascicle arrangements in computational models of skeletal muscles is important, especially when investigating three-dimensional muscle deformations in finite element models. However, obtaining high resolution *in vivo* measurements of fascicle arrangements in skeletal muscles is currently still challenging. This motivated the development of methods in previous studies to generate numerical representations of fascicle trajectories using interpolation templates. Here, we present an alternative approach based on the hypothesis of a rotation and divergence free (Laplacian) vector field behavior which reflects observed physical characteristics of fascicle trajectories. To obtain this representation, the Laplace equation was solved in anatomical reconstructions of skeletal muscle shapes based on medical images using a uniform flux boundary condition on the attachment areas. Fascicle tracts were generated through a robust flux based tracing algorithm. The concept of this approach was demonstrated in two-dimensional synthetic examples of typical skeletal muscle architectures. A detailed evaluation was performed in an example of the anatomical human tibialis anterior muscle which showed an overall agreement with measurements from the literature. The utility and capability of the proposed method was further demonstrated in other anatomical examples of human skeletal muscles with a wide range of muscle shapes and attachment morphologies.

## Introduction

Skeletal muscles have a wide range in anatomical architectures that reflects their diversity in functions [1], [2]. Because of their complex organization, skeletal muscle shapes often display heterogeneous curvatures, while the tendon and bone attachment areas have varying morphologies, ranging from broad surfaces to narrow central insertions. Inside each muscle, contraction and mechanical force are generated by muscle fibers, which are elongated contractile cells embedded in a matrix of connective tissue. Although the fiber cells typically do not have the length to span the entire muscle, they are grouped in parallel bundles called fascicles, which run between the proximal and distal sites where the muscle attaches to tendon structures or to bones. Hence, the muscle shape and morphology of the attachments are important determinants of the intramuscular arrangement of the fascicles.

Because the mechanical action of a skeletal muscle is transmitted along the fascicle tracts, it is important to obtain an adequate quantification of their trajectories when constructing computational models of muscle mechanics. Skeletal muscles are often modeled using a lumped-parameter approach that assumes a simplified arrangement of fascicles and tendons, e.g. [3], [4]. However, these simplified representations are not capable of capturing the three-dimensional (3D) deformations of muscles which have complex fascicle arrangements. For this purpose, volumetric modeling based on continuum mechanics [5] is better suited, in combination with the finite-element (FE) method to solve the numerical equations [6].

Finite-element modeling of skeletal muscle is challenged by the diversity of fascicle arrangements across all skeletal muscles. In order to accurately capture the mechanics of the muscle, the models' representations of the shape and fascicle arrangement must be anatomically realistic. However, fascicle trajectories are difficult to measure experimentally. Measurements based on dissection of cadaver specimens, e.g. [7]–[9], are mostly limited to a few sparse locations in the muscle while only a few studies have conducted a detailed dissection, e.g. [10], [11]. Ultrasound imaging is now frequently used for *in vivo* measurements because of its fast acquisition time and accessibility, e.g. [12], [13]. Despite these advantages, current techniques are predominantly limited to the visualization of the projected pennation angle in the two-dimensional (2D) image plane (B-mode imaging), which cannot image the whole muscle and is sensitive to the probe orientation [14], [15]. Diffusion tensor magnetic resonance imaging (DT-MRI) is currently the only three-dimensional technique that is capable of visualizing fascicle trajectories within any muscle in the body, e.g. [16]–[19]. However, the tracing algorithms that are used to reconstruct the trajectories are not always robust because of the noise in the acquired imaging data. Hence, tuning of constraint parameters or post-processing based on additional exclusion conditions is often necessary to remove tracts that are considered to be erroneous, e.g. [16], [18].

In order to circumvent the paucity of direct measurements of *in vivo* fascicle trajectories, FE muscle models use a variety of approaches to represent arrangements of fascicles. Blemker and Delp [20] developed a method in which fascicle orientations are numerically constructed based on fascicle map templates on a unit cube FE mesh that is warped to fit the muscle geometry as reconstructed from MRI data. The templates are created by defining control points according to the morphology of the tendon attachments, while a 3D spline interpolation gives the distribution of fascicle orientations. This approach has been shown to be useful for a range of muscle types but requires construction of multiple templates depending on tendon morphology, e.g. [20], [21]. Other studies have also proposed spline-based methods, but using reported measurements of superficial fibers as input for the interpolation [22]–[24], while demonstration of feasibility was often limited to a specific muscle. These previously proposed methods contain procedures to tailor templates or interpolation parameters for specific muscles, which can become impractical in concrete applications and makes it difficult to reproduce the computational model.

Although interpolation methods offer a pragmatic solution to obtain computational representations of fascicle arrangements, they do not explicitly take into account common architectural patterns as observed in many skeletal muscles. Incorporating these typical characteristics by a mathematical description could offer a theoretical alternative that can cover a wide variety of muscle types and which can also be fine-tuned by matching with experimental observations for a specific muscle. Therefore, we propose an alternative physics based approach to construct distributions of fascicle orientations in skeletal muscles. The method is based on the observation that fascicle trajectories have the following properties: (i) they are co-axially aligned and hence do not cross each other, (ii) they do not branch, (iii) they will not reverse their directions abruptly, and (iv) they must connect between the tendon or bone attachments to convey mechanical action, which means that they only originate or terminate in areas of origin (proximal) and insertion (distal). Based on these observations, we hypothesize that fascicle arrangements in skeletal muscles can be mathematically represented by a rotation and divergence free Laplacian vector field. To address this hypothesis, we: 1) developed a numerical framework to calculate fascicle trajectories based on the Laplace equation, 2) evaluated the resulting fascicle trajectories in a human tibialis anterior muscle through comparison with *in vivo* DT-MRI data as reported in the literature, and 3) demonstrated the general capability of the method in a number of examples of upper- and lower-limb muscles with a diverse range of shapes and attachment morphologies.

## Materials and Methods

### Theoretical foundation

The rotation free condition implies that the vector field representing the fascicle orientations is conservative and as such can be described as the gradient of a potential field 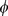. To meet the divergence free condition, 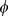 must be a solution of the Laplace equation:
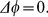
(1)


**Figure 1:**
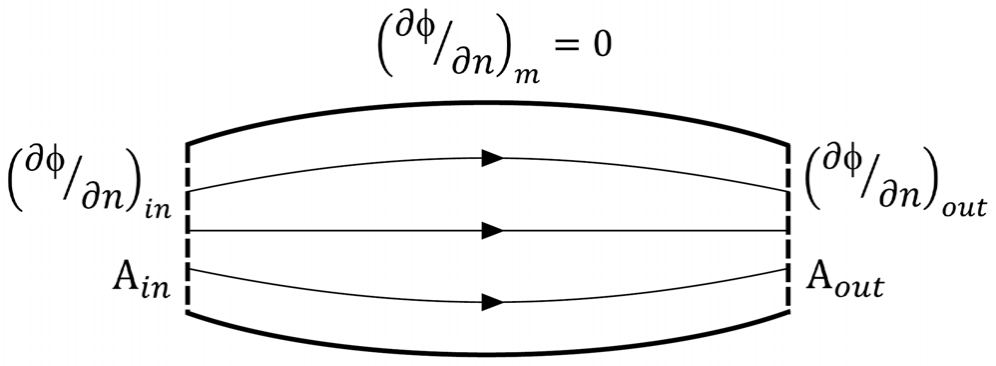
Overview illustrating the boundary conditions. The Neumann boundary conditions imposed on the different regions of the muscle surface to solve the Laplace equation (1) are shown. The full lines represent the surface of the muscle belly, while the tendon attachment regions are indicated by dashed lines. The potential gradients normal to the surface are denoted by 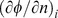, while 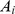 indicates the area of the tendon attachment surfaces.

Solutions of the Laplace equation are determined by the muscle geometry and the boundary conditions that are imposed. In case of standard Dirichlet and Neumann boundary conditions, it can be shown that the solution is unique [25]. In Neumann conditions, the potential gradient normal to the boundary surface or flux, 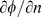, is imposed while this is the potential itself in Dirichlet conditions. The boundary conditions imposed to calculate the Laplacian vector field were defined as follows (Figure 1). Since fascicle tracts can only originate and terminate at the origin and insertion attachments, the potential gradient at the boundary surface of the muscle belly, 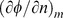, must be zero. At the attachment surfaces with areas 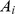, we hypothesize that a uniform flux as boundary condition will result in realistic muscle fascicle trajectories. Hereto, an inflow flux is defined at one tendon attachment area, which is balanced by an outflow flux at the other such that the mass equation is respected:
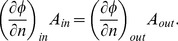
(2)


Since only the orientations of the vectors are relevant, the magnitude of the boundary flux can be arbitrarily chosen. Because the Laplace equation is linear, the solution will only be scaled by the flux magnitude while reversing the boundary fluxes will only change the direction but not the orientation of the vectors. This is analogous to a steady potential flow in fluid dynamics, where the potential and potential gradient represent the pressure and flow velocity respectively.

### Numerical workflow

#### Mesh construction

Three-dimensional muscle surface geometries were reconstructed by segmentation of static MR images as described in [20]. The VTK (v5.6.0) library (www.vtk.org) was used to extract the muscle surfaces as triangulated polygon meshes in the STL format. For each muscle, the attachment areas were identified and extracted as separate surfaces. Some minor regional smoothing was also performed in the Meshlab (v1.3.1) software (meshlab.sourceforge.net) to reduce artificial edges due to the slicing in the MR images. The polygon surfaces describing the muscle and attachment areas were used as input to construct tetrahedral volume meshes using the Gmsh (v2.6.0) software [26] with the following procedures. The STL surface triangulations were compounded and remeshed using an algorithm based on harmonic maps [27]–[29]. The muscle volume was subsequently filled with linear tetrahedral elements using the MMG3D anisotropic meshing algorithm [30]. A non-uniform element size was defined such that elements were about a factor 2 to 3 smaller at the attachment areas to reduce numerical noise in the fascicle tracing as described below. The magnitude of the element size was chosen such that the volume mesh contained roughly 300.000 linear elements.

#### Calculation Laplacian field

The Laplace equation (1) was solved by means of the finite-volume (FV) method. In this method, a solution is found for the potential field through iterative approximations of flux values across the mesh element faces, which must be balanced by the Laplacian of the potential 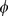 in the elements [31]. The finite-volume calculations were performed using the benchmarked OpenFOAM (v2.1.0) software (www.openfoam.org) which was combined with the pythonFlu (v2.1.0) wrapper software (pythonflu.wikidot.com) to allow for interactive scripting of the OpenFOAM algorithms in Python. The muscle meshes created in Gmsh were converted into native OpenFOAM mesh files which were combined with the default input files of the potentialFoam solver. The Neumann boundary conditions were imposed on the boundary faces of the muscle mesh. Since only the potential gradient was defined in the boundary conditions, the potential was set to an arbitrary value in a randomly chosen mesh element for numerical consistency. In the potentialFoam solver, the linear system of the discretized Laplace equation was solved by means of a preconditioned conjugate gradient (PCG) method combined with a symmetric diagonal incomplete Cholesky (DIC) preconditioner. The relative tolerance of the PCG algorithm was set to 1e-8, while the algorithm was iterated to correct for the non-orthogonality of the flux approximations until the two-norm of the residual vector dropped below 1e-8. The potential gradient representing the fascicle orientation vectors are calculated from the flux and potential field solutions in a post-processing step in the potentialFoam solver. The computation time on a standard desktop computer was estimated to be 500 seconds on average for the meshes considered.

#### Fascicle tracing

Muscle fascicle tracts were traced in the flux field by applying a streamline tracing method for divergence free flow fields as described by Klausen et al. [32]. In this approach, barycentric coordinates are used for an element-wise tracing which is controlled by the given flux values at the faces of each element. Because a divergence free flow field is incompressible, the sum of the fluxes 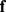 is zero in each element and it can be shown that in linear tetrahedral elements, the constant velocity vector (or potential gradient representing the fascicle orientation vector) 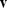 can be uniquely expressed in barycentric coordinates as:
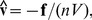
(3)with n the number of dimensions and V the element volume. The element intersection time 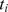 for each barycentric coordinate 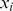 is given by:
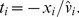
(4)


The time of flight 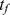 to traverse the element is given by the minimum non-negative intersection time. Hence, if the barycentric coordinates of the entry point is given by 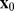, the exit point is calculated as:
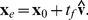
(5)


In this way, the path followed by the fascicle trajectory in each element can be determined from the time of flight 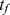. A more detailed description can be found in [32]. Because the tracing is performed element by element and is consistent with the given flux values, this method is very robust such that invalid terminations inside the muscle volume or at the muscle surface are avoided. However, the resulting fascicle tracts are locally non-smooth because of the linear discretization. Hence, the fascicle tracts were subsampled and a piecewise parametric spline smoothing was applied which was followed by a resampling to obtain 100 equidistant points for each fascicle tract.

The algorithms were implemented in Python (v2.7) (www.python.org) using the NumPy (v1.6.1) and SciPy (v0.9.0) numerical libraries (www.numpy.org). The correctness of implementations was verified by solving the Laplace equation in a parallelepipedon with boundary conditions as described above. Three-dimensional visualization was performed in MayaVi (v4.2.0) (Enthought Inc., Austin), while two-dimensional examples were visualized in Matplotlib (v1.1.0) (www.matplotlib.org).

### Case studies

First, the concept of the proposed method was demonstrated in synthetic 2D geometries which were constructed to represent a wide variety of typical skeletal muscle architectures. Second, anatomical 3D geometries were considered for examples of human muscles with a diverse range in architecture: tibialis anterior, Iliacus, gluteus maximus, adductor magnus, rectus femoris, vastus lateralis and deltoid. For the tibialis anterior example, fascicle lengths and pennation angles were compared with reported values in the literature. Hereto, fascicle length was calculated as the sum of Euclidean distances between fascicle tract points, while the procedure as described by Heemskerk et al. [18] was used to calculate a pennation angle on the surface of the distal tendon attachment. In this approach, the angle between the tangent plane in the seed points and vectors formed by the seed point and five consecutive tract points (which constitutes 5% of the fascicle length) was calculated. These five angles were subsequently averaged to obtain a measure of the pennation angle. The tangent plane in each seed point was determined by the average normal vector of the mesh face containing the seed point and its neighboring faces.

## Results

**Figure 2:**
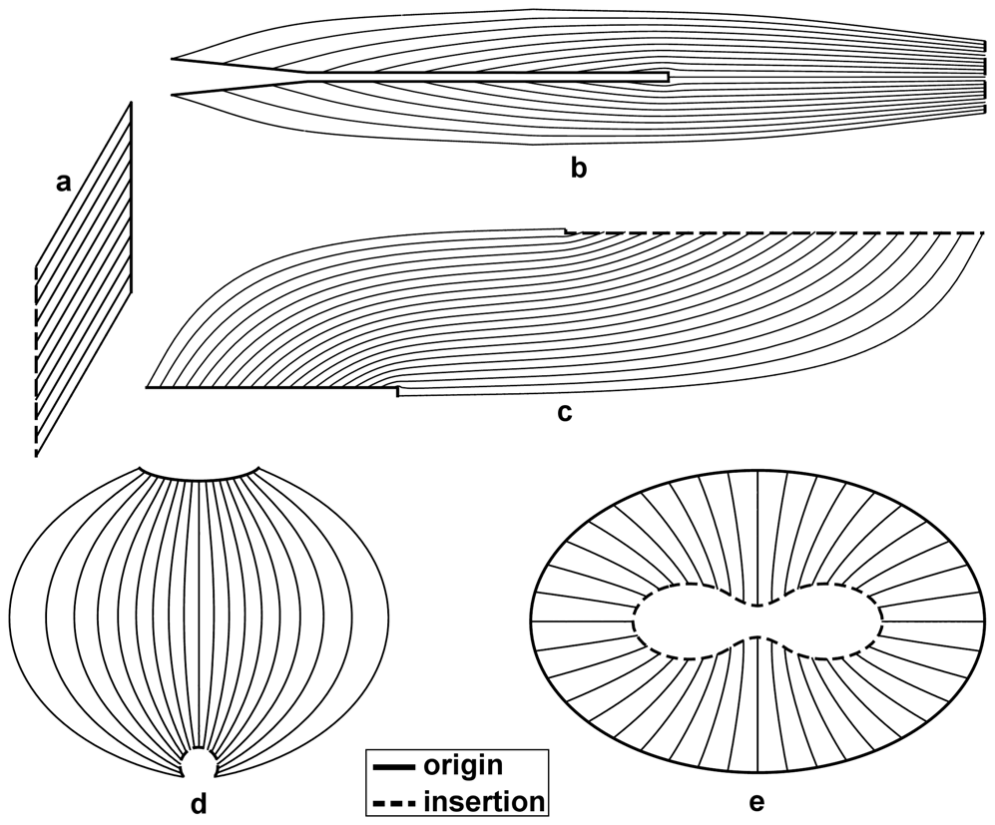
2D examples of calculated fascicle trajectories. These results obtained in 2D synthetic muscle geometries demonstrate the feasibility for complex shapes that are representative for anatomical muscle architectures: a simple pennate structure (a), long and narrow attachment as in the rectus femoris muscle (b), asymmetric broad attachments as in the biceps femoris muscle (c), a deltoid-like architecture (d) and a representation of the pectoral diaphragm (e).

The calculated fascicle trajectories in the synthetic 2D examples (Figure 2) are representative of the diversity in complexity and asymmetry that can be expected in skeletal muscle architectures. The morphologies of the origin and insertion attachments of a same muscle are often very different, posing a substantial challenge to construct a fascicle arrangement that has a natural distribution but still follows the shape of the muscle. Therefore, the 2D examples were constructed to test the proposed method under these challenging conditions. The results show that the generated fascicle trajectories have a plausible distribution even for these non-trivial geometries (Figure 2). Moreover, the method also allowed for generation of fascicle trajectories in muscles with a radial fascicle arrangement as for example in the pectoral diaphragm (Figure 2e).

**Figure 3:**
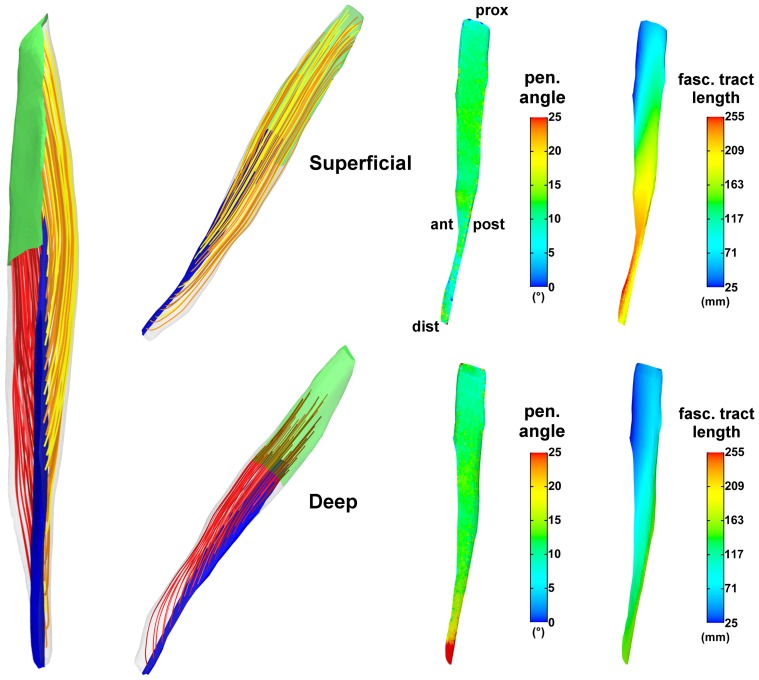
Results for the 3D anatomical example of the human tibialis anterior muscle. The muscle in this example is located in the lower left leg, anterior to the tibia and fibula. On the left, a sampling of the generated fascicle tracts is shown in an anterior view of the muscle. The origin (proximal) and insertion (distal) attachments are indicated in green and blue respectively. The insertion tendon (blue) divides the muscle in a deep and superficial compartment, of which the fascicle tracts are shown in red and yellow color tones respectively. The color tones are only used for purpose of enhancing the visual contrast. The fascicle tracts are shown in an anterolateral view for the two compartments separately in the middle column. On the right, the distributions of the calculated pennation angles and fascicle tract lengths are shown on the opposing sides of the insertion tendon surface in a similar fashion as in [17]. The upper and lower rows show the results for the superficial and deep compartments respectively. The anatomical orientations (ant = anterior, post = posterior, dist = distal, prox = proximal) are indicated for the superficial surface in the upper row and are the same for the deep surface.

The fascicle trajectories determined for the tibialis anterior muscle (Figure 3) display a good overall agreement with the measurements reported in the DT-MRI study by Heemskerk et al. [17]. They have found average pennation angles ranging between 6 and 22 with higher values in the superficial than in the deep compartment and with a general trend to increase in the distal-to-proximal direction. Their reported average fascicle lengths ranged between 50 and 160 mm and were greater in the deep than in the superficial compartment, increasing in the proximal-to-distal direction and decreasing in the anterior-to-posterior direction. A similar range in pennation angle was obtained in this study (Figure 3), with also higher values in the superficial than in the deep compartment although this difference is less pronounced than reported in [17]. However, at the distal end, higher pennation angles were obtained in the deep compartment. The fascicle lengths obtained in this study showed similar variations on the insertion tendon surfaces as reported in [17], although the range in values was larger while fascicle lengths were greater in the superficial than in the deep compartment.

**Figure 4:**
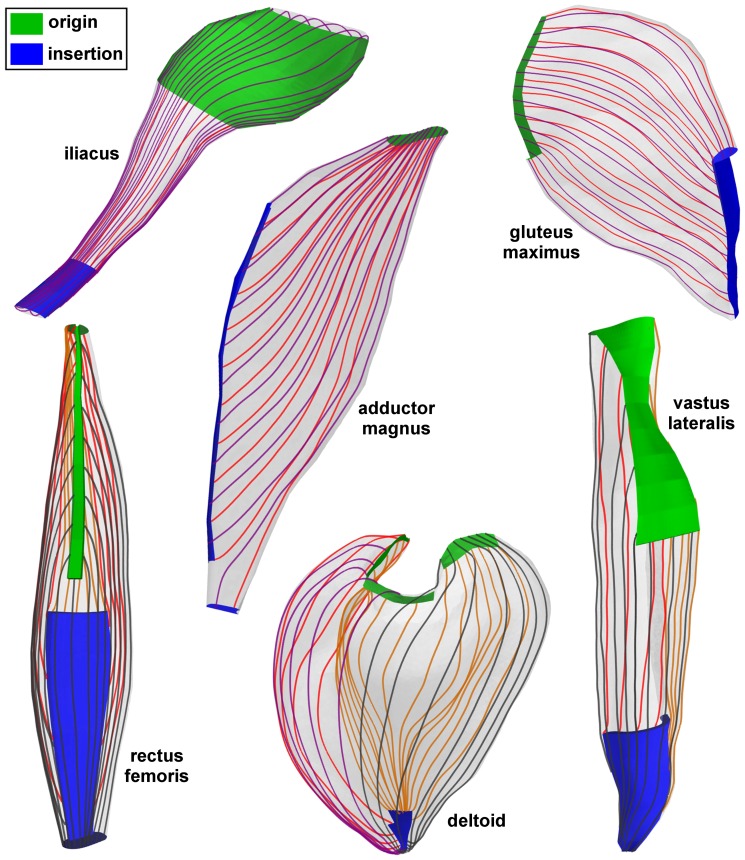
Generated fascicle trajectories in 3D anatomical examples of skeletal muscles. In each muscle, the origin (proximal) and insertion (distal) attachment regions are shown in green and blue respectively. For sake of visibility, only samplings of fascicle tracts on the muscle surface are shown. The different colors of the fascicle tracts are only for purpose of visual contrast.

The 3D results (Figure 4) demonstrate the potential of the proposed method to generate fascicle trajectories for a variety of skeletal muscles, despite the high level of architectural complexities, confirming the test results from the 2D examples. These anatomical examples encompass the following complexities: broad shape and attachments (iliacus and gluteus maximus), fanned shape (adductor magnus), fusiform with a long narrow tendon and broad attachment (rectus femoris) and wrapped shape (deltoid and vastus lateralis). Typical surface fascicle patterns as described in anatomical reference works, e.g. [1], [2], were obtained for each of the muscles: curved in the iliacus and in the gluteus maximus, fanned in the adductor magnus, bipennate in the rectus femoris, fanned and curved in the deltoid and oblique in the vastus lateralis.

## Discussion

The goal of this study was to develop and evaluate a computational methodology to generate representations of fascicle arrangements in skeletal muscle models that reflect observed physical characteristics of fascicle trajectories. To achieve this goal, a numerical approach based on the Laplace equation with standard Neumann boundary conditions was proposed and tested with a range of 2D and 3D examples illustrating a wide variety of muscle shapes and fascicle arrangements. The results support the idea that fascicle trajectories generally follow a Laplacian field and that the proposed method can provide a useful general method for defining fascicle trajectories for a wide range of skeletal muscles. The concept of the proposed method was demonstrated in 2D examples with synthetic geometries that contain asymmetries between origin and insertion attachment shapes often found in skeletal muscles (Figure 2).

A further evaluation was performed using a 3D anatomical surface reconstruction of the *in vivo* human tibialis anterior muscle as test case. To allow for a volumetric comparison, we compared calculated values with DT-MRI measurements, which are three-dimensional and give a detailed distribution of values in the *in vivo* muscle volume, contrary to the average or sparse measurements as usually obtained in dissection and ultrasound studies. The comparison of the results in the human tibialis anterior example with experimental data from [17] showed that the proposed method generates physiologically realistic fascicle trajectories (see Figure 3) that were generally similar to DT-MRI results. While overall our tibialis anterior results were similar to those reported by Heemskerk et al. [17], a few subtle differences did exist, which could be due to a number of factors. First, the DT-MRI tracking algorithm used by Heemskerk et al. [17] was unable to produce correct traces in the outermost distal region, which was therefore not considered in their analysis; whereas our approach reconstructed fascicle trajectories in the entire muscle volume. Second, the calculation of the pennation angle is sensitive to the normal vector on the tendon surface and positions of the sampled trace points, which are quantified differently in our tibialis anterior model. Third, variations in anatomy between the data sets could contribute to differences in muscle lengths and fascicle lengths. Fourth, user defined length and curvature based stop criteria were used in the DT-MRI fiber tracking in [17], whereas all fascicles traverse the entire muscle volume from origin to insertion in our method.

Our proposed method was further investigated in other anatomical examples of human muscles with a large variety of complex muscle shapes and attachment morphologies to demonstrate its utility and robustness. Unfortunately, there is a paucity of experimental measurements of volumetric *in vivo* 3D fascicle trajectories in the literature, as the majority of DT-MRI studies of skeletal muscles focus on the analysis of imaging parameters such as fractional anisotropy or diffusion coefficient, while tractography results were mostly shown for visual appreciation only, e.g. [16], [33]. Our results rendered plausible fascicle trajectories in all muscle examples that were considered, which demonstrates promise of the broad applicability of the technique.

The results of our study demonstrate that in the case of the tibialis anterior muscle, the proposed Laplacian approach can generate a fascicle arrangement that agree well with physiological observations based on DT-MRI measurements, despite the challenging morphology of the attachments in this muscle. The study by Heemskerk et al. [17] shows that the trends in observed spatial characteristics can be reproducibly detected. Given that the main characteristics of the shape and attachment structures (proximally broad, distally narrow central tendon) in the tibialis anterior are similar between individuals, it can be expected that the Laplacian field representation will give similar agreements in models of other subjects. For purpose of a subject-specific biomechanical analysis for which models would be created from in-vivo MRI data, DT-MRI measurements of fascicle orientations is the most complementary source that gives volumetric data without the need of invasive manipulation which can influence the measurements. This allowed for a thorough comparison of the spatial distributions of fascicle properties in our model of the tibialis anterior muscle. The results for the other examples that we have considered were not verified in as much detail, but illustrates that a Laplacian based approach can offer a common theoretical basis to capture the general characteristics of fascicle arrangements in other muscles, especially when experimental data is not available. Further validation would be required to confirm these initial results, which can be obtained through comparison with detailed *in vivo* DT-MRI measurements that quantify volumetric fascicle orientations, lengths and pennation angles in a diversity of skeletal muscles. In addition, detailed dissection studies in cadaveric specimens such as [11] can also be used for this purpose to verify the agreement between both data sources.

Solving the differential Laplace equation (1) to generate fascicle orientations requires a definition of the boundary conditions at the attachment areas (as illustrated in Figure 1). In this study, it was assumed that a uniform flux as boundary condition at the attachment areas was a suitable choice. This approach allowed for a simple matching of the inflow and outflow fluxes as described by equation (2), which can be easily imposed in standard solvers such as OpenFOAM. Alternatively, non-uniform flux distributions can be used as boundary conditions at the attachment areas to match measurement data of pennation angle or fascicle lengths. This requires a parametrical description of the flux distributions of which the parameters can be determined through an iterative optimization routine based on a best-fit criterion. Implementation would be achieved by an optimization approach in which the Laplace equation (1) is solved repeatedly in the iteration steps. The use of the Laplace equation has been recently considered in a similar application context. Levin et al. [34] designed an algorithm to denoise DT-MRI data based on a Helmholtz-Hodge decomposition of the muscle fiber vector field which also imposes the divergence and rotation free constraints, resulting in the Laplace equation as a penalty function. However, the behavior of the fiber vectors at the attachment areas were not defined by boundary conditions but by adding a non-zero divergence term in the vector field decomposition. They have visually demonstrated good results based on a DT-MRI data set of the forearm, which supports the concept of using the Laplace equation as a basis to generate representations of fascicle arrangements in skeletal muscles.

The Laplace based approach described in this study has several unique advantages. A computational representation of fascicle arrangements can be constructed when direct measurements are absent or unfeasible, but can be adapted through optimization to match with volumetric or sparse measurement data. The same approach can be applied to muscles with different architectures, as illustrated in Figure 4. Moreover, it is capable of generating fascicle trajectories for multiple tendon attachment sites as demonstrated in the adductor magnus and deltoid examples (see Figure 4). Hence, the need to modify templates or interpolation functions for specific muscles as in other methods [20], [22]–[24] is avoided, which incites computational reproducibility. It should be noted that few muscles such as the pectoralis major are characterized by twisting fascicles [28], which are most likely unfeasible to reproduce based on the Laplace equation because of the rotation free condition. However, we believe that the fascicle arrangements in the majority of skeletal muscles will follow the Laplacian conditions.

In conclusion, we have presented and evaluated an alternative approach to computationally model fascicle trajectories in skeletal muscles as divergence and rotation free Laplacian vector fields. Application in an example of the human tibialis anterior muscle showed an overall agreement with in-vivo DT-MRI measurements. In addition, feasibility and robustness of the method was demonstrated in examples of several skeletal muscles with a diverse range in anatomical architectures. The proposed approach offers the possibility to model fascicle arrangements in skeletal muscles with complex geometries and multiple attachment sites based on the differential Laplace equation. Further refinement and optimization of boundary conditions need to be investigated, as well as the feasibility for muscles with complex attachment morphologies such as the soleus muscle [24]. Future studies that extend the conceptual ideas presented in this paper can make use of the shared code and models that we have posted on “simtk” (https://simtk.org/home/musclefib).
